# Sickness absence in gender-equal companies A register study at organizational level

**DOI:** 10.1186/1471-2458-11-548

**Published:** 2011-07-11

**Authors:** Ann Sörlin, Ann Öhman, Lars Lindholm

**Affiliations:** 1 Department of Public Health and Clinical Medicine, Centre for Global Health Research, Umeå University, Sweden; 2 Umeå Centre for Gender Studies, Umeå University, Sweden

## Abstract

**Background:**

The differences in sickness absence between men and women in Sweden have attracted a great deal of interest nationally in the media and among policymakers over a long period. The fact that women have much higher levels of sickness absence has been explained in various ways. These explanations are contextual and one of the theories points to the lack of gender equality as an explanation. In this study, we evaluate the impact of gender equality on health at organizational level. Gender equality is measured by an index ranking companies at organizational level; health is measured as days on sickness benefit.

**Methods:**

Gender equality was measured using the Organizational Gender Gap Index or OGGI, which is constructed on the basis of six variables accessible in Swedish official registers. Each variable corresponds to a key word illustrating the interim objectives of the "National Plan for Gender Equality", implemented by the Swedish Parliament in 2006. Health is measured by a variable, days on sickness benefit, also accessible in the same registers.

**Results:**

We found significant associations between company gender equality and days on sickness benefit. In gender**-**equal companies, the risk for days on sickness benefit was 1.7 (95% CI 1.6-1.8) higher than in gender**-**unequal companies. The differences were greater for men than for women: OR 1.8 (95% CI 1.7-2.0) compared to OR 1.4 (95% CI 1.3-1.5).

**Conclusions:**

Even though employees at gender-equal companies had more days on sickness benefit, the differences between men and women in this measure were smaller in gender-equal companies. Gender equality appears to alter health patterns, converging the differences between men and women.

## Background

This register**-**based study evaluates possible connections between company gender equality and sickness absence from work. Swedish research on gender differences in sickness absence has shown that women have higher levels of sickness absence, especially long-term absence [1]. These differences in sickness absence have been a topic of considerable debate in the national media over a long period, but explanations for the differences have varied. Between 2000 and 2002 the most common explanation was that sickness absence was mainly due to unhealthy work practices and poor adaptation of the working environment to women by employers. In the period 2003-2004 there was a shift, with the most common explanation for the gender difference now being; overutilization of the insurance system and system disorders due to the medicalization of labour politics and regional politics [2]. The medicalization of politics was found to imply that workers were sick listed instead of being reported as unemployed. The explanation for some of the gender differences was also that women are more likely to take sickness absence when they have problems of a personal nature. In the current study, we use *sickness absence *as an overall term, whilst *sickness leave *refers to the first 2-14 days of sickness absence and *sickness benefit *to the period from day 15 onwards. Here we have looked exclusively at sickness benefit, as these data are available in public registers used in this study.

All types of inequities in the distribution of health in a population are of major concern to public health and of major interest to public health research. Sex differences in health are well known, and the gender system is often considered one of many important social determinants of health [3-5]. There are at least two main routes when discussing gender differences: one is that biology is the main reason for the differences, the other is that they are due to major differences in the lives of men and women [6-10]. In the current study, we evaluate the possible impact of a lack of gender equality at work on health. Biological differences are not discussed in this paper.

In a global perspective, women live longer than men, but are more often sick, have reduced opportunities for education and paid employment, lower social status in families, communities and society, they have limited access to and control over resources, limited decision-making power, and are more exposed to sexual and gender-based violence as a result of unequal gender norms [11].

Gender theory research has highlighted the existence of strong societal structures that can be summarized by the term *gender order*. Gender order is characterized by a disassembled relationship between what is considered feminine and masculine, and of a hierarchy in which the phenomena and characteristics associated with men are usually held higher than those perceived as female. Even if the specific terms that signals male or female change over time, but the gender order is reproduced [12-15].

The idea of promoting health by increasing gender equality is not new [8,16]. The United Nations, too, believes that increased gender equality can promote social equity. Further, in 2005, 170 heads of state set time-bound targets - the Millennium Development Goals - ranging from halving extreme poverty to promoting gender equality [17]. However, the idea of gender equality as a self**-**evident goal for all might be an overly narrow view. A Swedish survey of public health workers showed that different systems of belief play important roles in the judgment of fairness and desire for change regarding differences in health between men and women [18].

### Sickness absence

Sickness absence in Sweden rose between 1997 and 2007, with women accounting for the major part of the increase. Since 2007 there has been a decrease, with the decrease for women greater than for men [19]. There are various reasons for the decrease, but two important factors are believed to be the composition of insurance and benefits on the one hand and people's attitudes to sickness absence on the other. Individuals act in accordance with the normative (behaviour) views of their social network [20-23]. Other studies [24] have emphasized the interplay of working life and private circumstances. Differences in health between men and women and the relation between gender differences and the number of days on sick leave have been investigated in previous studies. The gendered construction of health has been found to relate to different patterns in the practice of ill health and the outcome of sick leave for men and women [25,26]. How sickness absence is measured may therefore be important for the gender differences found [27].

Targeting research to individuals and to individual conditions can make it difficult to capture structural conditions and risks masking underlying conditions. Susan Philips, a well-known researcher in public health, called for an index to assess the impact of gender on wellbeing. She proposed a group-level measure of equality between men and women [28]. We similarly developed an index to measure differences in gender equality at company level on a large scale. The index is based completely on data available in public registers, and in this study we have chosen a health measure (sickness benefit) also available in the same registers. The nature of the study is of necessity explorative since there is very little research in this area. However, examining the literature, two hypotheses, here used as general presumptions, can be formulated:

• Gender equality influences health through convergence. The differences today are largely caused by the gender order, and when this order is abolished, the differences will decrease. Men's mortality will decrease while morbidity will increase, and vice versa for women. This theory of convergence implies that sick leave taken by men will increase in gender**-**equal companies compared to gender-unequal companies, and vice versa for women.

• The theory of expansion of roles implies that women in gender**-**equal companies will have a greater total workload as they are promoted to more advanced roles in the companies at the same time as they continue to carry out the greater part of household work. This leads to extra strain on women in gender**-**equal companies compared to women in unequal companies. Thus, this hypothesis would predict more days on sick leave for women in gender**-**equal companies.

If we summarize the two assumptions above, suggesting contradictory lines of development, a time**-**dependent hypothesis emerges. In the short term expansion of roles dominates as the change in household division of labour has been known to appear later than women's entrance into the labour force. In the long run, we believe that people working in gender**-**equal companies will take less days on sickness absence than those working in gender**-**unequal companies, although the data available now characterize more of the short**-**term perspective.

### Research questions

The present study seeks to answer the following research questions. Does increased gender equality reduce the numbers of days on sickness benefit at company level? Is the impact of gender equality at work different for men and women?

## Methods

To evaluate the impact of gender equality on health at company level, we used the OGGI index [29] to measure gender equality and days on sickness benefit to measure health.

The OGGI index is a policy driven index that is aimed at evaluating the compliance to the "National Swedish Plan for Gender Equality" adopted by the Swedish Parliament in 2006 [30]. The overall aim of the policy is:

*Women and men shall have equal power to shape society and their own lives. A prerequisite to accomplish this is that women and men enjoy the same opportunities, rights and obligations in all spheres of life*.

**Figure 1:**
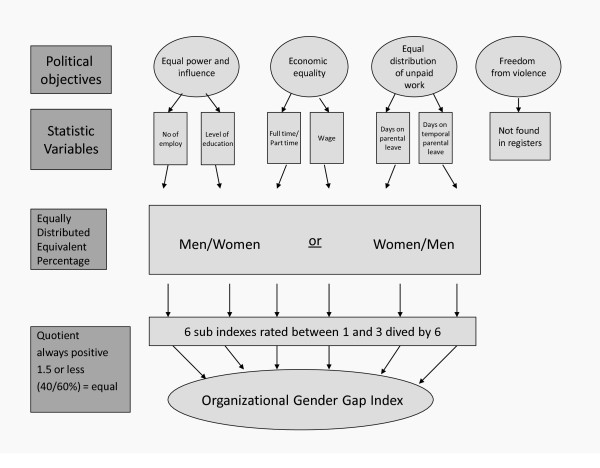
**Construction of the index**.

The first three of the four interim goals of the plan were transposed to key words as follows: 1) equal power and influence 2) economic equality of men and women and 3) equal distribution of unpaid work. These were connected to six variables in the official registers as outlined in Figure 1.

### Data collection

Data were collected from two public registers**: **(i) *LISA*, the *Longitudinal Integration Database for Social Insurances and Labour Market Studies*, and (ii) *WSD*, *Wage Statistics Descriptions*. The LISA database contains individual information on demographic data, education, employment, family, income and so on. The LISA database forms the official basis for Swedish longitudinal statistics and research. The database presently holds annual registers from 1990 and includes all individuals from 16 years of age who where registered in Sweden on December 31 for each year. More information on the work place such as number of employees at the company, occupational grade, employment type and sector were obtained from the WSD database. All data used in this study are from 2004. In the LISA database, the prime focus is the individual. It can be linked to other databases, such as Wage Statistics which is a company-level database. The LISA database also contains information on sickness benefit. The database is updated every year and the registers are publicly available after 17 months, except for the data on workplaces which are available after 20 months. Work on the Organizational Gender Gap Index (OGGI) began in 2006; therefore, the data used in this study derive from statistics available at Statistics Sweden from 2004. To avoid data bias, we have used information on sickness benefit from the same year.

The variables used in the study were the following: male/female employee ratio, percentage of full**-**time employees, educational level in years, monthly income, days on parental leave, and days on temporary parental leave. The means at company level, divided by sex, were calculated for each variable. In the index, each variable is presented as a quota calculated irrespective of the favoured sex. A ratio of 40/60 was regarded as equal. The six variables constitute the Organizational Gender Gap Index [29].

Two sectors were chosen for the application of the OGGI. The first was the Computer sector. Here, we excluded the part of the sector relating to repair and machinery support. In total, 19,551 persons in 46 companies from this sector were included in the study. The second sector chosen was the Grocery production sector. All kinds of grocery production, including for instance animal slaughter, food production, industrial baking and production of alcohol, are part of this large sector. In total, 33,733 persons in 77 companies belonged to this sector in 2004. The Swedish labour market is strongly segregated along gender lines and in the private sector fewer than 20% of employees are women. In order to enable the intended comparisons between men and women as well as between companies within a sector, we chose parts of the private sector that we knew employed both women and men despite this segregation. The Computer sector was labelled a white-collar sector and the Grocery production sector a blue-collar sector. The choice of sectors was influenced by earlier research revealing differences in working environment, employment and earnings, sickness absence and health complaints [24,31,32].

The chosen construction of the index resulted in removal of companies with only male or female employees, or with fewer than 10 employees. In total, the index comprised 53,204 persons in 123 companies.

### Sickness absence

In Sweden, sickness absence is registered differently depending on whether it is the first period, days 2 till 14 of the sickness absence called "days on sick pay", or the second period, day 15 and over, termed "days on sickness benefit". For the first period, sickness absentees are paid by their employer and statistics are maintained by the Swedish Social Insurance Administration. The second period is paid by the health insurance office and these statistics are available in both the Social Insurance Administration and Statistics Sweden's official registers. In this study, we used the statistics from Statistics Sweden's register, i.e. "days on sickness benefit".

We chose sickness benefit (the period from day 15 and over) for two reasons. The first was because the initial period includes many single and two-day absences which can be due to colds and other seasonal factors. We thus argued that the long-term absence would be more related to problems at the workplace. The second reason was that we aimed to find a model that would be cost effective and efficient for those working with it, usually Human Resources (HR) personnel. Using one single register for the study turned out to be cost effective.

The sickness benefit variable was dichotomized at individual level: either a person had been on sickness benefit or not. At company level, means were calculated separately for the numbers of men and the numbers of women on sickness benefit.

### Analysis and calculations

Logistic regression analyses were conducted to assess associations between the OGGI index for the company and the mean at company level for the numbers of days on sickness benefit. The analyses were also adjusted for age, education, income, full**-**time**/**part**-**time employment and business sector. All the data analyses were conducted using PASW Statistics 18 (formally known as SPSS Statistics).

### Ethical approval

For the register study Statistics Sweden's Board of Ethics approved the retrievals from the databases. Data were delivered in aggregated form as statistical retrievals and the individual data were anonymous.

The research project was also approved by the Regional Ethics Review Board in Umeå, number 07-124 M.

## Results

### Main findings

**Figure 2:**
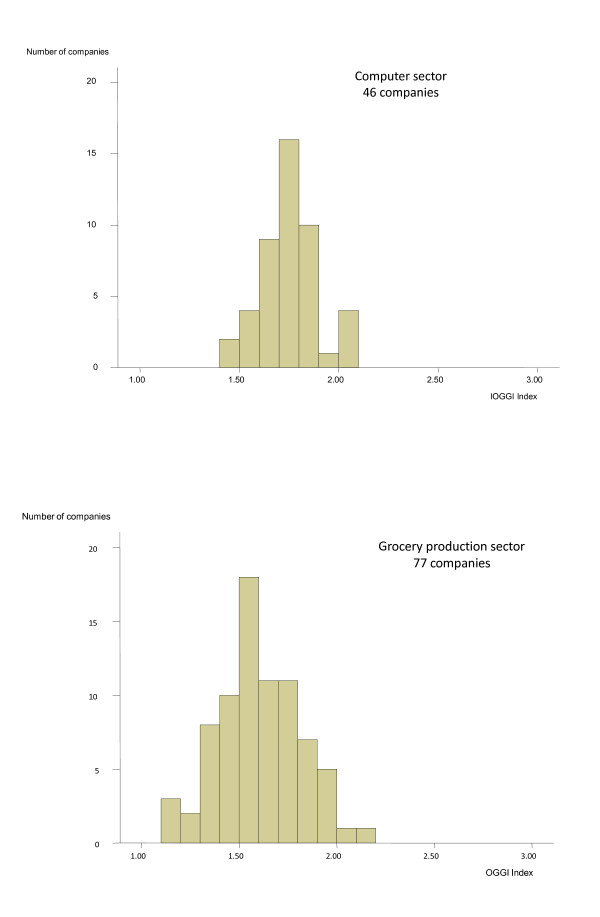
**Distribution of index by company in the Computer sector and Grocery production sector**.

As shown in Figure 2 only 22% of the companies were ranked as gender equal with an index score between 1.13 and 1.5. The remainder of the companies were ranked as unequal, with index scores between 1.51 and 2.14.

Table 1 presents the distribution of the variables divided by sector, sex and gender equality. In the Grocery production sector, almost as many women work in gender**-**equal companies as in unequal companies. For men in this sector, the number in unequal companies is almost twice the number in equal companies. In the Computer sector, the difference is even greater, with only 385 of 14,023 male respondents working in gender**-**equal companies and a similar pattern for women, namely 174 of 5,424. Income disparity is greater in the Computer sector than in Grocery production sector. In the Computer sector, men take twice as many days of parental leave (mean 6.17 days) as men in the Grocery production sector (mean 3.03 days). For women the difference between the sectors is almost threefold: a mean of 21.18 days in the Computer sector compared to a mean of 8.7 days in the Grocery production sector. The number of days of parental leave is higher in gender**-**equal companies in both sectors, for both women and men.

**Table 1:** Description of the variables divided by sector, sex and GE

	Computer sector		Grocery production sector	
	**Men**	**Women**	**Men**	**Women**

***No***.	14023	5598	23092	10686
Gender equal	385	174	7102	4068
Unequal	13638	5424	15990	6618

***Income mean***	38,020	32,215	24,587	21,995
Gender equal	32,849	27,748	24,221	21,493
Unequal	38,168	32,358	24,750	22,303

***Parental leave days mean***	6.17	21.81	3.03	8.7
Gender equal	9.02	30.14	4.12	7.25
Unequal	6.09	21.54	2.94	9.65

***Temporary parental leave days mean***	1.45	1.92	1.38	1.98
Gender equal	1.92	2.41	1.72	2.27
Unequal	1.44	1.90	1.23	1.29

***Full/part time mean***	0.99	0.96	0.95	0.92
Gender equal	0.99	0.97	0.98	0.95
Unequal	0.99	0.96	0.92	0.90

***Education*** ***years mean***	15.40	15.38	12.66	12.76
Gender equal	15.31	15.60	12.48	12.45
Unequal	15.50	15.37	12.74	12.95

***OGGI mean***	1.70	1.69	1.62	1.58
Gender equal	1.46	1.47	1.41	1.38
Unequal	1.71	1.70	1.71	1.70

***Mean age***	41.00	39.65	39.55	40.20
Gender equal	36.73	36.47	39.16	40.31
Unequal	41.12	40.07	39.72	40.14

There are significant associations between gender equality and days on sickness benefit at company level (see table 2). In gender**-**equal companies, the risk for days on sickness benefit is 1.7 (95% CI 1.6-1.8) higher than in gender**-**unequal companies. The differences are greater for men than for women**: **1.8 (95% CI 1.7-2.0) compared to 1.4 (95% CI 1.3-1.5).

**Table 2:** Association between the gender equality index and sickness benefit

Variables	Sickness benefit			
	
	Men		Women	
	
	No	Yes	No	Yes
Type of company, n (%)				
Gender-equal companies	6639 (88.8)	835 (11.2)	3461 (81.8)	772 (18.2)
Gender-unequal companies	27669 (93.6)	1890 (6.4)	10380 (86.4)	1640 (13.6)
*Unadjusted OR (95% CI)*				
Gender-equal companies	1.8 (1.7-2.0)		1.4 (1.3-1.5)	
Gender-unequal companies	1		1	
Adjusted OR (95% CI) *				
Gender-equal companies	1.4 (1.3-1.5)		1.3 (1.1-1.4)	
Gender-unequal companies	1		1	

Note:*adjusted for age, education, income, full**/**part time and sector

In the logistic regression, days on sickness benefit as a dichotomized variable was used as the outcome. In the Computer sector, 93% of employees had no days on sickness benefit as measured in this study; in the Grocery production sector, the figure was 88.5%. We found an expected difference in days on sickness benefit between men and women in both sectors. The ratios between men and women are lower for gender**-**equal companies in both sectors and the difference is greater in the Computer sector. It seems that women's days on sickness benefit are less negatively affected by gender equality than men's, and this could possibly be a result of the convergence theory. The ratios between men and women presented in table 3 are for the Computer sector 2.15 for gender**-**equal companies and 3.23 for unequal companies. For the Grocery production sector, the ratio for gender**-**equal companies is 1.82 and for unequal companies 2.22.

**Table 3:** Mean number of days on sickness benefit and ratio of women to men

	Computer sector			Grocery Production sector		
	**Women**	**Men**	***Women/men ratio***	**Women**	**Men**	***Women/men ratio***

*Gender equal companies*	21.11	9.83	**2.15**	17.18	9.41	**1.82**
*Gender unequal companies*	16.31	5.05	**3.23**	15.04	6.76	**2.22**

## Discussion

Differences in days on sickness benefit, both between men and women and between the sectors, are expected. The increase in days on sickness benefit in gender**-**equal companies is hardly good news, and supports the theory of expanded roles[33] regarding women. The increase in men's days on sickness benefit in gender**-**equal companies supports the convergence hypothesis. The convergence theory [3,34] implies that gender equality will affect men and women differently. According to this theory, with greater gender equality men will show increased morbidity and reduced mortality, with the opposite outcome for women. The difference in days on sickness benefit found in this study could also be an effect of a change in the normative system regarding what is accepted as a reason for absence. Perhaps gender**-**equal companies are forerunners and allow a more caring atmosphere, which could encourage men to acknowledge poor health and consequently take greater sick leave. The classic masculine stereotype is of denying signs of sickness up to the point of very serious illness, which may partly explain higher male mortality.

A study of Swedish municipalities found that gender equality correlated with poorer health for both men and women [8]. This result agrees with the present study, but we also found that the differences between men and women are smaller in gender**-**equal companies. Other research has shown that the relationship between gender equality and health depends to a great extent on sex, life sphere and type of inequality. This thus seems rather to require a combination of the convergence and stress expansion theories [35]. To understand the findings, the study also requires more nuanced explanations than the convergence theory alone can provide.

The number of days on sickness benefit is decreasing in the total population for various reasons [36], and the differences between men and women are converging. We claim that such a development would be beneficial for both men and women. If parental obligations were shared equally by couples, women would be able to pursue careers similar to those of men, which would be beneficial for women in terms of lifetime income levels, pensions and career development. Men, on the other hand, would benefit if they were more attentive towards their own ill health, staying at home when sick instead of going to work. These changes in behaviour regarding health/ill health might also alter the gendered patterns of mortality in the long term and lead to a convergence between men and women [34].

The definition of gender equality varies between studies, making it difficult to make objective comparisons. Here we used an interpretation of Swedish national policy as our definition. There are reasons to believe that this definition is somewhat top down, in particular regarding parental leave and temporary parental leave. A significant number of people argue that the family itself should decide the mother's and father's share of parental leave, and that many different allocations could be "gender fair". For the present index, only variables available in public registers were used. We might therefore have omitted some important issue or issues, or something in the index should perhaps not be included. However, this is a necessary trade**-**off: studies on a larger scale would be extremely expensive if they used questionnaires or interviews, and are thus not likely to be undertaken. We believe that the register information, however imperfect, is valuable and should be utilized.

Temporary parental leave has been shown to be a good proxy for gender division in the household [37]. Although it is one of the variables in our index, a wider perspective is of course required when measuring gender equality at work. There is an interdependence between the household and workplace conditions, as the household division of gender equality of course also affects the individual at work, and vice versa. Thus companies alone cannot be blamed for shortcomings in the division of parental leave; however, they do have a responsibility to facilitate families to make a reasonable division. Companies successful in this respect unambiguously deserve a high grade on any measurement of gender equality in the workplace.

Other social determinants/conditions can also have affected our results. In Sweden, young women have a higher average educational level than men, with 48% of women between 25 and 44 years of age having completed higher education. The corresponding figure for men is 37% [38]. In our study, it is only in the gender-equal companies in the Computer sector that women have a higher education level than men. Another factor is the large difference in the number of days of parental leave taken by employees in the Computer sector and the Grocery production sector. Especially for women, where we find more than double the number of days taken in the Computer sector (21.81 days compared to 8.7 in the Grocery sector), this is probably due to the fact that women with higher education tend to have their children later in life[39]. However, this could of course also be due to less possibilities of staying at home with children in a sector where the average income is low, or to other reasons not observed in this study.

Our outcome measure "days on sickness benefit" has known shortcomings as a measure of health. Besides the individual's health, social norms and regulations also strongly influence the level of days on sickness benefit. The available data were truncated to episodes longer than 15 days. Total sickness absence days are likely a more appropriate and sensitive measure in this kind of study. The reality behind the expansion of roles theory is daily time constraints causing frictions in the family that probably have a negative impact on both short and long**-**term illness.

## Conclusions

Although there are significant associations between company gender equality and days on sickness benefit, and even though the risk for sick leave is greater in gender**-**equal companies than in gender**-**unequal companies, the ratios between men and women in the Computer sector are 2.15 for gender**-**equal companies and 3.23 for unequal companies. For the Grocery production sector, the ratio for gender**-**equal companies is 1.82 and for unequal companies 2.22. This could be due to a convergence between men and women of patterns of days on sickness benefit in gender-equal companies, suggesting that gender equality will affect men and women differently. If the differences in health patterns between men and women can be influenced by increased gender equality, this could be a useful instrument for HR officers and policymakers in the future. The findings of this study could help improve our understanding of the complex patterns that result in differences between men's and women's sickness absence. The OGGI index could become a useful tool for future monitoring of changes in gender relations at company level.

## Competing interests

The authors declare that they have no competing interests.

## Authors' contributions

AS is the corresponding author and is also responsible for the database and calculations. AS, LL and AÖ outlined and wrote the article together. All authors read and approved the final manuscript.

## Authors' information

Institutional address:

Umeå University, Dept of Public Health and Clinical Medicine, Epidemiology and Global Health, SE-901 87 Umeå, Sweden

## Pre-publication history

The pre-publication history for this paper can be accessed here:

http://www.biomedcentral.com/1471-2458/11/548/prepub
